# Kaurenoic Acid from *Aralia continentalis* Inhibits Biofilm Formation of *Streptococcus mutans*

**DOI:** 10.1155/2013/160592

**Published:** 2013-04-04

**Authors:** Seung-Il Jeong, Beom-Su Kim, Ki-Suk Keum, Kwang-Hee Lee, Sun-Young Kang, Bok-Im Park, Young-Rae Lee, Yong-Ouk You

**Affiliations:** ^1^Jeonju Biomaterials Institute, Jeonju 561-360, Republic of Korea; ^2^Wonkwang Bone Regeneration Research Institute, Wonkwang University, Iksan 570-749, Republic of Korea; ^3^Department of Pedodontics, School of Dentistry, Wonkwang University, Iksan 570-749, Republic of Korea; ^4^Department of Oral Biochemistry, School of Dentistry, Wonkwang University, Iksan 570-749, Republic of Korea; ^5^Wonkwang Research Institute for Food Industry, Iksan 570-749, Republic of Korea

## Abstract

We isolated a single chemical compound from *A. continentalis* and identified it to be kaurenoic acid (KA) and investigated the influence of anticariogenic properties. Inhibitory effects of KA on cariogenic properties such as growth, acid production, biofilm formation, and the adherence of *S. mutans* were evaluated. Furthermore, real-time PCR analysis was performed to evaluate the influence of KA on the genetic expression of virulence factors. KA significantly inhibited the growth and acid production of *S. mutans* at 2–4 **μ**g/mL and 4 **μ**g/mL of KA, respectively. Furthermore, the adherence onto S-HAs was inhibited at 3-4 **μ**g/mL of KA and biofilm formation was significantly inhibited when treated with 3 **μ**g/mL KA and completely inhibited at 4 **μ**g/mL. Also, the inhibitory effect of KA on biofilm formation was confirmed by SEM. In confocal laser scanning microscopy, bacterial viability gradually decreased by KA in a dose dependent manner. Real-time PCR analysis showed that the expressions of *gtfB, gtfC, gbpB, spaP, brpA, relA*, and *vicR* were significantly decreased in *S. mutans* when it was treated with KA. These results suggest that KA from *A. continentalis* may be a useful agent for inhibiting the cariogenic properties of *S. mutans*.

## 1. Introduction

Dental caries, which are also known as tooth decay or cavities, constitute one of the most common forms of ecological disease of the teeth. Most caries are caused by the dissolution of the teeth, by acid produced from specific type of bacteria, oral bacteria [1, 2].

Dental caries are caused by some types of oral streptococci. Among these bacteria, *Streptococcus mutans *(*S. mutans*) is the most important cariogenic bacteria [3, 4]. Dental caries are formed in 2 steps. The first step involves the initial attachment of the oral bacteria such as *S. mutans *to the tooth surface. *S. mutans *synthesizes an insoluble glucan layer via glucosyltransferase (GTFase), and it is a well-known factor that accelerates the maturation of dental plaque [4, 5]. In the second step, *S. mutans* metabolizes the carbohydrates contained in consumed foods and produces organic acids, which initiates the dissolution of the tooth enamel. The accumulation of acid in the dental plaque leads to decalcification and breakdown of the teeth and can result in the formation of dental caries [6]. 

Fluoride compounds have been used as antiplaque. Despite fluoride being effective against dental plaque formation, high levels of fluoride are cytotoxic [7, 8]. Thus, many attempts have been made to search for an effective agent to prevent dental plaque formation [9, 10]. Recently, several natural products that are derived from herbs, such as *Rosmarinus officinalis*, *Mentha longifolia* L., *Aralia continentalis,* and *Curcuma longa L.*, have also been studied for their ability to inhibit the formation of dental plaque [11–14]. 

*A. continentalis* is a perennial plant and a member of the Araliaceae family. It is cultivated extensively in Asia, Siberia, China, and Korea and has a long tradition of use in Korean traditional medicine to reduce pain and inflammation [15, 16]. In addition, *A. continentalis* has been used to treat dental diseases such as toothaches and periodontal disease [17]. Recently, we have reported that a crude extract from *A. continentalis* inhibits the *S. mutans* bacterial growth, acid production, adherence to hydroxyapatite beads HAs, and biofilm formation [14]. However, there was no evidence of a specific antibacterial single compound because various compounds were contained in the crude extract.

Therefore, in the present study, we isolated a single compound from the extract of *A. continentalis *and identified it as kaurenoic acid (KA:* ent*-kaur-16-en-19-oic acid). Then we examined that influence of KA on the growth, acid production, bacterial attachment, and biofilm formation of *S. mutans, *and several virulence factor expressions, which are associated with dental plaque and caries formation, were assessed.

## 2. Materials and Methods

### 2.1. Plant Material

Plant material in the form of roots of *A. continentalis *was collected in Imsil, Korea, in November 2004 and identified and authenticated by Professor Young-Seung Ju at the College of Oriental Medicine, Woosuk University. A voucher specimen (No. JSI 53) was deposited at the College of Oriental Medicine, Woosuk University, Jeonju, Korea.

### 2.2. Isolation and Identification of Compounds

**Figure 1:**
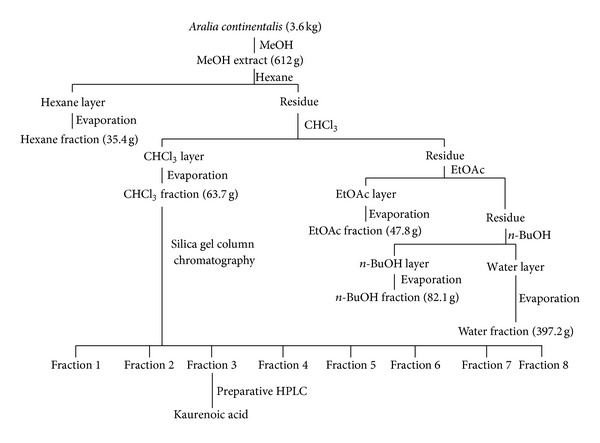
Fraction flow chart of kaurenoic acid isolation from *Aralia continentalis*.

**Figure 2:**
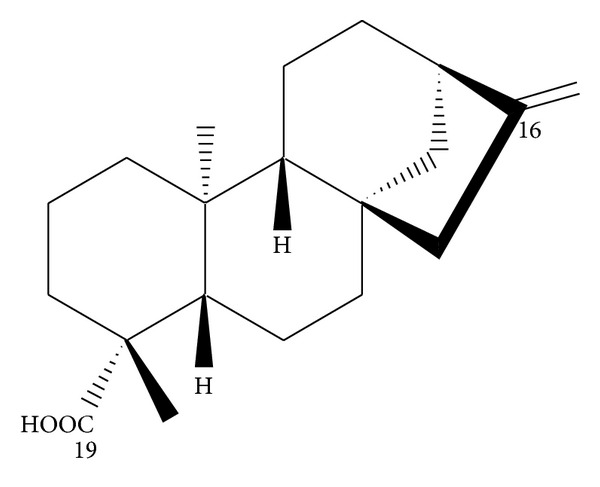
Structure of isolated compound: kaurenoic acid (KA;* ent*-kaur-16-en-19-oic acid).

The dried roots of *A. continentalis* (3.6 kg) were crushed and extracted with methanol (MeOH) (18 L) under reflux for 4 h. The extract was then concentrated under reduced pressure to afford an extract (612 g, 17%), which was suspended in H_2_O and successively partitioned with normal hexane (*n*-hexane), chloroform (CHCl_3_), ethyl acetate (EtOAc), and *n*-butyl alcohol (*n*-BuOH) that had been saturated with H_2_O. Each solvent was evaporated *in vacuo* to yield an *n*-hexane fraction (35.4 g, 0.9%), CHCl_3_ fraction (63.7 g, 1.7%), EtOAc fraction (47.8 g, 1.3%), *n*-BuOH fraction (82.1 g, 2.3%), and H_2_O fraction (397.2 g, 10.8%). The CHCl_3_ layer (59.6 g) was subjected to silica gel column chromatography of increasing polarity (hexane-EtOAc, 100 : 0 → 1 : 1) to give fractions 1–8. Fraction 3 (5.6 g) was purified by preparative HPLC (LC-9104, JAI ODS-BP-L column, MeOH, 3.5 mL/min, and detection at 254 nm) to obtain a pure active component (739.2 mg, tr = 58.2 min). This compound was identified as being kaurenoic acid (KA;* ent*-kaur-16-en-19-oic acid) by comparing its spectral data (MS, ^1^H- and ^13^C-NMR) with those reported in the literature [15, 18] (see Figure 1). KA: white powder; mp 178-179°C; [*α*]_D_^25^−110° (*c*1.0, CHCl_3_); IR*v*_max⁡_ (KBr) cm^−1^ 3450 (OH), 1680 (C=O), 1470 (C=C); ^1^H-NMR (500 MHz, CDCl_3_) *δ* 0.83 (2H, dd, *J* = 13.2, 4.2 Hz, H-1), 0.92 (3H, s, H-20), 1.08 (1H, m, H-5), 1.21 (3H, s, H-18), 1.5 (4H, m, H-7 and 12), 1.62 (2H, m, H-11), 2.09 (2H, d, *J* = 3.0 Hz, H-6), 2.61 (1H, br s, H-13), 4.72 (1H, br s, H-17_a_), 4.77 (br s, H-17_b_); ^13^C-NMR (125 MHz, CDCl_3_): *δ* 41.38 (C-1), 19.19 (C-2), 37.85 (C-3), 43.92 (C-4), 57.16 (C-5), 21.91 (C-6), 41.38 (C-7), 44.31 (C-8), 55.18 (C-9), 39.78 (C-10), 18.52 (C-11), 33.20 (C-12), 43.85 (C-13), 39.74 (C-14), 49.05 (C-15), 155.91 (C-16), 103.14 (C-17), 29.07 (C-18), 185.10 (C-19), 15.68 (C-20). Chemical structure of KA is shown in Figure 2. 

### 2.3. Effect of KA on Bacterial Growth

*S. mutans *was purchased from American Type Culture Collection (ATCC; Rockville, MD, USA). To evaluate the inhibitory effect of KA on bacterial growth, *p*-iodonitrotetrazolium violet (INT; Sigma-Aldrich, St. Louis, MO, USA) colorimetric assay was used as described previously [14]. Briefly, the bacteria were cultured in 0.95 mL of brain heart infusion (BHI: Difco, Detroit, MI, USA) broth that contained 1% glucose and various concentrations of KA at 37°C under aerobic conditions. Then, *S. mutans* was cultured overnight and inoculated in 0.05 mL in BHI broth at a density of 5 × 10^5^ colony-forming units (CFU)/mL. After 24 h of cultivation, 0.2 mL of *p*-iodonitrotetrazolium violet (INT; Sigma-Aldrich, St. Louis, MO, USA) was added to each tube. Then bacterial growth was indicated by formazan produced after 2 h of incubation. The optical density of the INT formazan was measured at 550 nm using a spectrophotometer.

### 2.4. Effect of KA on Acid Production

Effect of KA on *S. mutans* acid production was determined by a previous method [14]. Briefly, KA solution was filtered using a 0.2 *μ*m syringe filter, and the solution was added to 0.95 mL of phenol red broth that contained 1% glucose. Then, *S. mutans* (5 × 10^5^ CFU/mL density) was inoculated and cultured at 37°C. After 24 h of cultivation, the pH of the bacterial growth media was directly measured using a pH meter (Corning Inc., Corning, NY, USA). To confirm the effect of KA solution on the pH, the initial pH of the media was also measured before inoculating it with *S. mutans*.

### 2.5. Measurement of Bacterial Adherence

Bacterial adherence was assessed according to a previous study [14] using a hydroxyapatite beads (diameter of 80 *μ*m; Bio-Rad, Hercules, CA, USA). Briefly, thirty micrograms of hydroxyapatite beads were coated with clarified human saliva [19] for 1 h at room temperature. Then the saliva-coated hydroxyapatite beads (S-HAs) were rinsed 3 times with 0.01 M potassium phosphate buffer (KPB; pH 7.0). The S-HAs were immersed in bacterial suspension (1 × 10^7^ CFU/mL) solution without and with various concentration of KA. To allow for bacteria adsorption, the S-HAs were gently agitated for 90 min at 37°C. Following this, the S-HAs were washed and transferred to a new tube that contained KPB. To disperse the adsorbed *S. mutans *onto the S-HAs, physical stress was applied using a sonicator (Fisher Scientific, Springfield, NJ, USA) at 50 W for 30 s. Then the supernatants were diluted and spread on MSA plates. After 48 h of cultivation at 37°C, the numbers of bacterial colonies were counted.

### 2.6. Effect on Biofilm Formation

Biofilm formation was assessed according to a previous study [20]. Various concentrations of KA were added to BHI broth that contained 0.1% sucrose in 35 mm polystyrene dishes, 24-well plates (Nunc, Copenhagen, Denmark), or 24-well plates that contained resin teeth (Endura, Shofu Inc., Japan). The cultures were created in the allotted broths by inoculating them with seed cultures of *S. mutans *(5 × 10^5^ CFU/mL) and allowed to grow for 24 h at 37°C. The supernatants were removed and the dishes or wells were rinsed with distilled water. Biofilm formation was measured by staining with 0.1% safranin. The biofilms that formed on the surface of the resin teeth were also stained with 0.1% safranin and photographed. Then, the bound safranin was eluted from the stained cells using 30% acetic acid and the supernatants were measured at 530 nm.

### 2.7. Scanning Electron Microscopy Observations

To evaluate biofilm formation, the biofilms that formed on the 35 mm polystyrene dishes were analyzed by performing scanning electron microscopy (SEM). The biofilms that formed on the dishes were rinsed with distilled water and fixed with 2.5% glutaraldehyde in 0.1 M sodium cacodylate buffer (pH 7.2) at 4°C for 24 h. The samples then were dehydrated in an ethanol gradient series (60%, 70%, 80%, 90%, 95%, and then 100%) and then freeze-dried. The samples were sputter-coated with gold and viewed by SEM (JOM-6360, JEOL, Tokyo, Japan).

### 2.8. Bactericidal Effect of KA

Bactericidal effect of KA was determined by confocal laser scanning microscopy. The cultured *S. mutans *in BHI was diluted using BHI media to approximately 1 × 10^7^ CFU/mL. The bacteria (1 × 10^7^ CFU/mL) were treated with various concentrations of KA at 37°C under aerobic conditions. After 24 h of incubation, the bacteria were washed with PBS and stained with LIVE/DEAD BacLight Bacterial Viability Kit (Molecular Probes, Eugene, OR, USA), prepared according to manufacturer's instructions, for 15 min. Stained bacteria were observed by confocal laser scanning microscopy (LSM 510, Zeiss, Germany). This method is based on two nucleic acid stains: green fluorescent SYTO 9 stain and red fluorescent propidium iodide stain which differ in their ability to penetrate healthy bacterial cells. SYTO 9 stain labels live bacteria; in contrast propidium iodide penetrates only bacteria with damaged membranes. 

### 2.9. Real-Time Polymerase Chain Reaction (PCR) Analysis

To determine the effect of KA on gene expression, a real-time PCR assay was performed. The subminimal inhibitory concentration (1–3 *μ*g/mL) of KA was used to treat and culture *S. mutans* for 24 h. Total RNA was isolated from *S. mutans* by using Trizol reagent (Gibco-BRL) according to manufacturer's instructions. Then, cDNA was synthesized using a reverse transcriptase reaction (Superscript; Gibco-BRL). The DNA amplifications were carried out using an ABI-Prism 7,000 Sequence Detection System with Absolute QPCR SYBR Green Mixes (Applied Bio-Systems Inc., Foster City, CA, USA). The primer pairs that were used in this study were described by a previous report [21] and are listed in Table 1. 16S rRNA was used as an internal control.

### 2.10. Statistical Analysis

All of the experiments were performed in triplicate. All of the values are expressed as a mean ± standard deviation. The statistical analysis was performed using Student's *t*-test and one-way ANOVA. A *P* value of <0.05 was considered to indicate statistical significance.

## 3. Results 

### 3.1. Bacterial Growth Inhibition by KA

**Figure 3:**
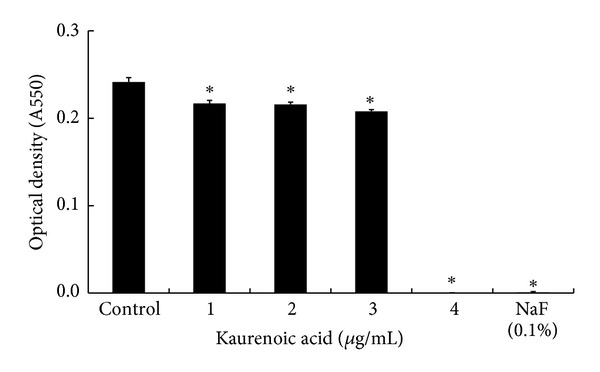
Effect of kaurenoic acid (KA) on *Streptococcus mutans *growth and adherence to saliva-coated hydroxyapatite beads. The bacteria were inoculated into BHI broth with various concentration of KA and cultured for 24 h at 37°C. Inhibitory activity is shown in the presence of KA at concentrations ranging from 1 to 4 *μ*g/mL. Each value is expressed as a mean ± standard deviation. Significance was determined at *P < 0.05 when compared with the control.

In this study, KA was isolated from *A. continentalis* as an active compound that exhibits anticariogenic effects. *S. mutans *was treated with 1, 2, 3, and 4 *μ*g/mL of KA, and subsequent bacterial growth was determined. As shown in Figure 3, KA (1–3 *μ*g/mL) significantly inhibited the growth of *S. mutans* (*P* < 0.05). In addition, bacterial growth was completely inhibited on using 4 *μ*g/mL of KA as it was in the positive control group (0.1% NaF). These results show that KA extracted from *A. continentalis* may inhibit the growth of *S. mutans*.

### 3.2. Inhibitory Effects of KA on Acid Production by *S. Mutans*

The inhibitory effect of KA on acid production by *S. mutans* was evaluated. *S. mutans *was cultured in the presence of various concentrations (1–4 *μ*g/mL) of KA, and the pH change was measured. As summarized in Table 2, the pH was significantly decreased at 5.03 ± 0.05 for the control group. When treated with 1–3 *μ*g/mL KA, the pH also decreased but not significantly. However, the pH decrease was significantly inhibited at 4 *μ*g/mL of KA and it was approximately similar to the positive group (0.1% NaF). These results indicate that KA that was extracted from *A. continentalis* may inhibit the production of organic acid by *S. mutans*.

### 3.3. Inhibitory Effect of KA on Bacterial Adherence and Biofilm Formation

**Figure 4:**
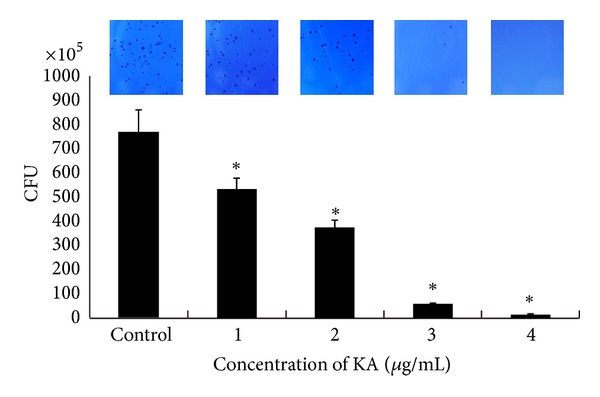
Effect of kaurenoic acid (KA) on colony-forming units (CFU). *Streptococcus mutans *was inoculated into BHI broth with various concentration of KA and cultured for 24 h at 37°C. Inhibitory activity is shown in the presence of KA at concentrations ranging from 1 to 4 *μ*g/mL. The CFU of *S. mutans* that adhered to 30 mg of saliva-coated hydroxyapatite beads that were treated with various concentration of KA are shown. When treated with 1–4 *μ*g/mL KA, adherence was markedly repressed. Each value is expressed as a mean ± standard deviation. Significance was determined at *P < 0.05 when compared with the control. NaF was used as a positive control.

We tested the inhibitory effect of KA on the ability of *S. mutans *to adhere to S-HAs. The adherence of *S. mutans *that had been cultured in the presence of KA was significantly inhibited in a dose dependent manner. In addition, adherence onto S-HAs was obviously inhibited at 3-4 *μ*g/mL of KA (Figure 4).

**Figure 5:**
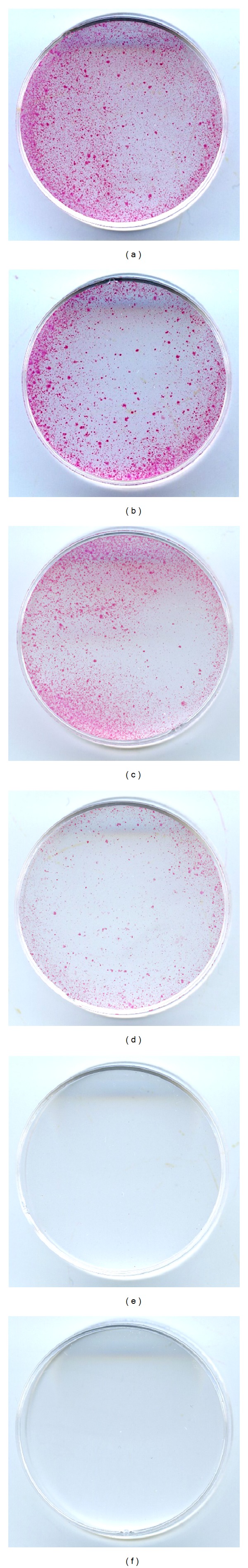
Effect of kaurenoic acid (KA) on *Streptococcus mutans *biofilm formation. *S. mutans* was inoculated into BHI broth with various concentrations of KA and cultured for 24 h at 37°C. The biofilms that formed on the dish surface were measured by staining with 0.1% safranin. Biofilm formation was significantly inhibited at 3 and 4 *μ*g/mL KA. (a) Control; (b) 1 *μ*g/mL, (c) 2 *μ*g/mL, (d) 3 *μ*g/mL, (e) and 4 *μ*g/mL of KA; and (f) positive control (0.1% NaF).

To determine whether KA that was isolated from *A. continentalis* inhibits the biofilm formation by *S. mutans*, bacteria were cultured in the presence of 1–4 *μ*g/mL of KA in polystyrene dishes. Then, the biofilm formation was observed by safranin staining. As shown in Figure 5, there was no significant inhibitory effect on biofilm formation at 1 and 2 *μ*g/mL KA. However, biofilm formation was significantly inhibited at 3 *μ*g/mL of KA and completely inhibited at 4 *μ*g/mL of KA.

**Figure 6:**
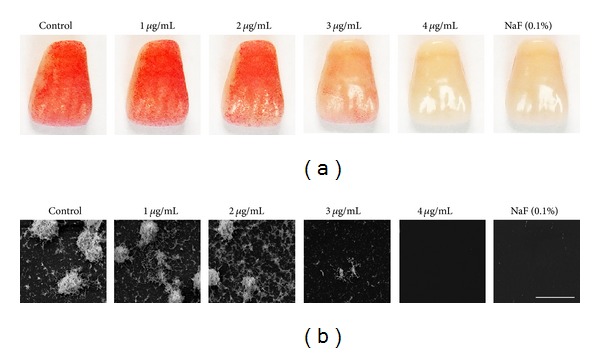
(a) The effect of kaurenoic acid (KA) on biofilm formation on resin tooth surfaces. *Streptococcus mutans *biofilms on resin tooth surfaces were incubated in various concentrations of KA. Biofilm formation was inhibited by treatment with 3 *μ*g/mL and completely inhibited at 4 *μ*g/mL KA. (b) Scanning electron microscopy image of *S. mutans* biofilm formation on resin tooth surfaces. NaF was used as a positive control. The scale bar represents 25 *μ*m.

Also, we observed biofilm formation on the surface of resion teeth by safranin staining. Treatment with 1 and 2 *μ*g/mL of KA slightly inhibited biofilm formation by *S. mutans*. However, *S. mutans* biofilm formation was significantly inhibited when treated with 3 *μ*g/mL of KA and completely inhibited at 4 *μ*g/mL (Figure 6(a)). In addition, the inhibitory effect of KA on biofilm formation was observed by SEM. The SEM image also showed results that were similar to those for safranin staining (Figure 6(b)).

**Figure 7:**
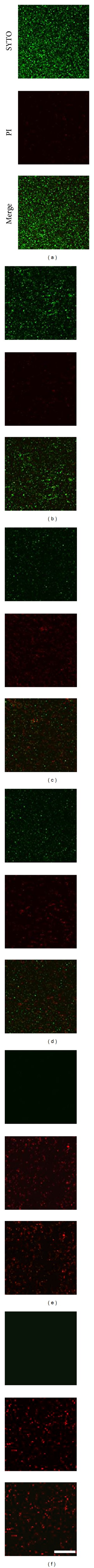
Bactericidal effect of kaurenoic acid (KA). Cultured *Streptococcus mutans* was treated with KA and stained with LIVE/DEAD BacLight Bacterial Viability Kit. The stained bacteria were observed by confocal laser scanning microscopy. Treatment with KA decreased green-labeled living bacteria (SYTO 9 stain) and increased red-labeled dead bacteria (PI stain) in a dose dependent manner. (a) Control; (b) 1 *μ*g/mL, (c) 2 *μ*g/mL, (d) 3 *μ*g/mL, and (e) 4 *μ*g/mL of KA; and (f) positive control (0.1% NaF). Bar = 50 *μ*M.

To determine bactericidal effect of KA, the cultured bacteria were stained with LIVE/DEAD BacLight Bacterial Viability Kit and observed by confocal laser scanning microscopy. Treatment with KA decreased green-labeled living bacteria (SYTO 9 stain) and increased red-labeled dead bacteria (PI stain) in a dose dependent manner (Figure 7). This result suggests that KA may have bactericidal effect on *S. mutans.*

### 3.4. Inhibitory Effect of KA on Virulence Factor Gene Expression

**Figure 8:**
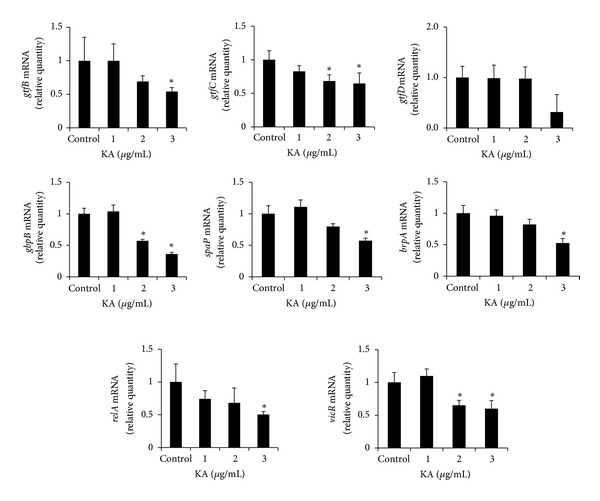
Real-time PCR analysis of expression of several virulence factor genes. *Streptococcus mutans *was cultured and treated with various concentrations of kaurenoic acid (KA) and real-time PCR analysis was then performed as described in Section 2. *gtfB*, *gtfC*, and *brpA *expressions were significantly inhibited at 3 *μ*g/mL of KA, but *gtfD *expression was not inhibited. *gbpB* and *spaP *were significantly inhibited at 2 and 3 *μ*g/mL of KA. The expression of *relA* was inhibited ranging over 1–3 *μ*g/mL KA. *vicR* expression was inhibited at 1 and 3 *μ*g/mL of KA. Each value is expressed as a mean ± standard deviation. Significance was determined at *P < 0.05 when compared with the control.

To evaluate the influence of KA on the gene expression of virulence factors in *S. mutans, *the bacteria were treated with subminimal inhibitory concentration (1–3 *μ*g/mL) of KA, and the gene expressions of virulence factors were assessed by real-time PCR (Figure 8). First, we evaluated the genetic expressions of *gtfB, gtfC*, and *gtfD*, which encode GTFase B, C, and D proteins, respectively. The expression of *gtfB* was significantly decreased when *S. mutans *was treated with 3 *μ*g/mL of KA, and *gtfC *was significantly decreased at 2 and 3 *μ*g/mL of KA. However, the expression of *gtfD* was not significantly affected by KA. The expressions of *gbpB* and *spaP*, which contribute to bacterial adherence, were also decreased at 2 and 3 *μ*g/mL of KA, respectively. The expressions of *brpA *and *relA, *which are related with acid tolerance, were significantly decreased at 3 *μ*g/mL of KA. *vicR, *which is associated with regulating the expressions of *gbp*B, *gtf*B, *gtf*C, and *gtf*D, was also decreased by KA treatment at the concentration of 2 and 3 *μ*g/mL.

## 4. Discussion

*A. continentalis *is reportedly used in traditional oriental medicine to treat pain and inflammation [15, 16]. Recently, we reported that an extract of *A. continentalis *has an antibacterial activity such as inhibiting bacterial growth, biofilm formation, and the adherence of *S. mutans *[14]. However, no single active compound that was responsible for those observations was determined. Therefore, in this study we isolated a single compound from *A. continentalis *and investigated its potential effects on the cariogenic properties of *S. mutans.*

We first investigated the growth inhibition activity of KA that was isolated from *A. continentalis* against *S. mutans*. To evaluate the antibacterial properties of numerous reagent or plant extractions, *S. mutans *was used because it is a major bacterium that is responsible for the formation of dental plaque and dental caries [3, 4]. These results showed that KA that was isolated from *A. continentalis *exhibits antibacterial activity by inhibiting the growth of *S. mutans*. 

*S. mutans* is found in human dental plaque and is one of the most cariogenic types of bacteria because it can metabolize dietary sugars and produce organic acids such as lactic acid and formic acid. Due to its metabolism, it can lower the pH of dental plaque, demineralize tooth enamel, and cause dental caries [6]. Therefore, any alternation in pH was taken to be an indicator for evaluating potential anticariogenic agents. In the present study, the inhibitory effect of KA that was isolated from *A. continentalis *on acid production by *S. mutans* was evaluated and it was found to be significant.

The ability to synthesize extracellular glucan from sucrose catalyzed by GTF is generally regarded as being a major factor in the creation of dental plaque by *S. mutans* [22]. Glucans induce bacterial adherence and result in the formation of biofilms [23]. Thus, to prevent dental plaque formation, inhibiting *S. mutans* adherence to the tooth surface is very important because adhesion is initially necessary process for dental plaque formation [5]. Herein, we tested the inhibitory effect of KA on the ability of *S. mutans *to adhere to S-HAs. Adherence of *S. mutans *that was cultured in the presence of KA was significantly inhibited. Also, we evaluated whether or not KA that was isolated from *A. continentalis *could affect biofilm formation by *S. mutans*. Our results showed that KA significanlty inhibited biofilm formation when treated with KA on polystyren dishes and the surface of resin teeth. This evidence indicated that KA directely altered the attachment and biofilm formation of *S. mutans*. 

To determine bactericidal effect of KA, the cultured bacteria were stained with LIVE/DEAD BacLight Bacterial Viability Kit and were observed by confocal laser scanning microscopy. The LIVE/DEAD BacLight Bacterial Viability assay is based on two nucleic acid stains: green fluorescent SYTO 9 stain and red fluorescent propidium iodide stain which differ in their ability to penetrate healthy bacterial cells. SYTO 9 stain labels live bacteria, in contrast propidium iodide penetrates only bacteria with damaged membranes. Treatment with KA decreased green-labeled living bacteria (SYTO 9 stain) and increased red-labeled dead bacteria (PI stain) in a dose dependent manner. This result suggests that KA may have bactericidal effect on *S. mutans.*

Several virulence factors are associated with cariogenicity in addition to adhesion and acid tolerance. Therefore, real-time PCR analysis was performed to evaluate the influence of KA on the gene expressions of virulence factors in *S. mutans*. 

Among its numerous virulence factors, 3 major genes, *gtfB, gtfC,* and *gtfD *(GTFase B,C, and D, respectively), are important virulence factors [21, 24]. GTFase synthesizes glucan polymer from sucrose, and synthesized glucans provide binding sites for bacterial adhesion on the tooth surface and contribute to the formation of dental plaque [25]. In a previous study, all 3 genes (*gtfB, gtfC,* and *gtfD*) appeared to be required for inducing maximal dental caries [26], but Aoki et al. [25] indicated that *gtfB* and *gtfC* are required for the attachment of *S. mutans* to hard surfaces. Besides these GTFase factors, *gbpB*, which encodes surface-associated glucan binding protein (GBP), is also an essential factor for *S. mutans* because it mediates cell-surface interaction with glucan polymer [27]. *S. mutans *possesses an *spaP *gene that produces SpaP protein, which acts as an adhesion by mediating adherence to salivary agglutinin-coated hydroxyapatite [24, 28]. In this study, KA significantly inhibited the gene expressions of *gtfB* and *gtfC* but not *gtfD*. In addition, the expressions of *gbpB* and *spaP* were also significantly inhibited by KA. Thus, our results suggest that one possible mechanism, in which KA inhibits the attachment of *S. mutans, may be *by repressing *gtfB*, *gtfC*, *gbpB*, and *spaP*.

In addition, *brpA* and *relA* have also been shown to play critical roles in environmental stress responses and biofilm development by *S. mutans*. *brpA *is associated with biofilm regulation [29], and *relA *gene plays a major role in several processes such as biofilm formation, glucose uptake, and acid tolerance [30, 31]. Shemesh et al. reported that *vicR *is a regulatory gene of other virulence factors in *S. mutans* such as *gbpB, gtfB, gtfC, and gtfD *[21]. Based on our results, the expressions of *brpA*, *relA*, and *vicR* were repressed by KA. This result indicates that KA may exhibit antibiofilm activity against *S. mutans *by inhibiting *brpA*, *relA*, and *vicR* expressions.

Our data showed that KA isolated from *A. continentalis *inhibited growth, adhesion, acid tolerance, and biofilm formation by *S. mutans* through the partial inhibition of several of its virulence factors. 

## 5. Conclusions

In the present study, we isolated a single compound from *A. continentalis* and identified the compound as being KA. In this study, we observed the inhibitory effects of KA on the growth, acid production, biofilm formation, and adherence of *S. mutans*. In addition, KA also inhibited the virulence factor gene expression in *S. mutans*. Our findings suggest that KA isolated from *A. continentalis* may have a potential as the chemical for preventing dental plaque formation by *S. mutans*. However, more biochemical and phytochemical investigations are necessary because dental plaque is produced through multiple regulatory systems.

## Figures and Tables

**Table 1:** Oligocucleotide primers that were used in this study.

Gene*	Primer sequences (5′-3′)	
*16S rRNA *	Forward	CCTACGGGAGGCAGCAGTAG
	Reverse	CAACAGAGCTTTACGATCCGAAA
*gtfB *	Forward	AGCAATGCAGCCAATCTACAAAT
	Reverse	ACGAACTTTGCCGTTATTGTCA
*gtfC *	Forward	GGTTTAACGTCAAAATTAGCTGTATTAGC
	Reverse	CTCAACCAACCGCCACTGTT
*gtfD *	Forward	ACAGCAGACAGCAGCCAAGA
	Reverse	ACTGGGTTTGCTGCGTTTG
*brpA *	Forward	GGAGGAGCTGCATCAGGATTC
	Reverse	AACTCCAGCACATCCAGCAAG
*gbpB *	Forward	ATGGCGGTTATGGACACGTT
	Reverse	TTTGGCCACCTTGAACACCT
*relA *	Forward	ACAAAAAGGGTATCGTCCGTACAT
	Reverse	AATCACGCTTGGTATTGCTAATTG
*spaP *	Forward	GACTTTGGTAATGGTTATGCATCAA
	Reverse	TTTGTATCAGCCGGATCAAGTG
*vicR *	Forward	TGACACGATTACAGCCTTTGATG
	Reverse	CGTCTAGTTCTGGTAACATTAAGTCCAATA

*Based on the NCBI *S. mutans* genome database.

**Table 2:** The pH of *S. mutans *cultures that were incubated with various concentrations of KA.

Conc. (*μ*g/mL)	pH (before incubation)	pH (after incubation)
Control	7.20 ± 0.05	5.03 ± 0.05
1	7.20 ± 0.05	5.00 ± 0.10
2	7.13 ± 0.05	5.00 ± 0.00
3	7.20 ± 0.05	5.13 ± 0.11
4	7.00 ± 0.00	6.97 ± 0.05*
0.1% NaF	7.20 ± 0.00	7.00 ± 0.00*

Each pH value is represented as mean ± standard deviation. *P < 0.05 when compared with the control group after incubation.
